# Supplementary material for: Introducing the Dutch Quality Registry for Acute Internal Medicine (DRAIM): Method of development and opportunities of use from a single-centre pilot study
Source: PLoS One. 2026 Jun 1;21(6):e0350110. doi: 10.1371/journal.pone.0350110 (PMC13225412; doi:10.1371/journal.pone.0350110)
Supplement: S1 Table — (DOCX) [file pone.0350110.s001.docx]

# Supplementary files

**Table S1. Dataset of the quality registry acute internal medicine**

| Data type | Variables recorded |
| --- | --- |
| *Patient characteristics* | |
| Demographics | - Age - Sex - Postal code - Living situation *(i.e. at home independently, at home with professional care, nursing home)* |
| Illness burden | - Age-adjusted Charlson Comorbidity Index (17) - Number of medications (actively prescribed) at home |
| Severity of illness in the ED | - Vital signs: systolic and diastolic blood pressure, pulse rate, respiratory rate, saturation derived from pulse oximetry, temperature, neurological state - Modified Early Warning Score (18) - Triage category *(i.e. Manchester Triage System, Netherlands Triage System or Boston Triage System)* |
| Laboratory findings in the ED and 48h after presentation | - Hematological: Hemoglobin, Mean Corpuscular Volume, Leukocytes, Thrombocytes - Chemistry: C-Reactive Protein, Creatinine, Urea, Sodium, Potassium, Albumin, Calcium, eGFR, pH, Base Excess, Lactate, D-dimer, Troponin-I |
| Type of disease | - Presenting complaint at presentation - (Working) Diagnosis: ICD-10 classification (19) |
| Other variables | - Hospital admission (<30 days) prior to ED visit - Way of referral to the ED *(i.e. self-referral, ambulance, referral by GP or referral by medical specialist)* |
| *Process measures* | |
| Hospital characteristics | - Type of hospital *(i.e. academic medical center, teaching hospital, general hospital)* - Number of beds - Number of beds in the Acute Medical Unit - Number of patients in hospital adherence area |
| Emergency Department characteristics | - Number of treatment bays - Number of shock rooms - Number of staff per shift - Number of patients - Number of admitted patients - Regional trauma function |
| Emergency Department visit characteristics | - Length of stay - Day and time of arrival - Number of medical specialties involved |
| *Outcome measures* | |
| Medical outcomes | - In hospital mortality at 7 and 30 days - Hospital admission - ED-revisits at 7 and 30 days - Hospital readmission at 7 and 30 days - ICU admission within 24 hours and during hospital admission |

*ED: Emergency Department. ICD-10: International Classification of Diseases, 10^th^ edition. ICU: Intensive Care Unit*
