# Supplementary material for: Introducing the Dutch Quality Registry for Acute Internal Medicine (DRAIM): Method of development and opportunities of use from a single-centre pilot study
Source: PLoS One. 2026 Jun 1;21(6):e0350110. doi: 10.1371/journal.pone.0350110 (PMC13225412; doi:10.1371/journal.pone.0350110)
Supplement: S2 Table — (DOCX) [file pone.0350110.s002.docx]

# Supplementary files

**Table S2. Top 5 presenting complaints in the ED.**

|  | All patients (≥65 years old)  (n=6701) | 65-74 years old  (n=2131) | 75-84 years old  (n=2500) | ≥ 85 years old  (n=1440) |
| --- | --- | --- | --- | --- |
| 1. | General malaise (n=2366, 35.3%) | General malaise (n=736, 36.1%) | General malaise (n=986, 41.3%) | General malaise (n=644, 46.6%) |
| 2. | Shortness of breath (n=750, 11.2%) | Fever (n=299, 14.7%) | Shortness of breath (n=319, 13.4%) | Shortness of breath (n=188, 13.6%) |
| 3. | Fever (n=724, 10.8%) | Shortness of breath (n=243, 11.9%) | Fever (n=280, 11.7%) | Fever (n=145, 10.5%) |
| 4. | Stomach ache (n=336, 5.0%) | Stomach ache (n=158, 7.8%) | Stomach ache (n=128, 5.4%) | Lower extremity complaints (n=55, 4.0%) |
| 5. | Lower extremity complaints (n=301, 4.5%) | Lower extremity complaints, (n=120, 5.9%) | Lower extremity complaints (n=126, 5.3%) | Stomach ache (n=50, 3.6%) |

*Missing* n=265 (4.0%).
