# Supplementary material for: Introducing the Dutch Quality Registry for Acute Internal Medicine (DRAIM): Method of development and opportunities of use from a single-centre pilot study
Source: PLoS One. 2026 Jun 1;21(6):e0350110. doi: 10.1371/journal.pone.0350110 (PMC13225412; doi:10.1371/journal.pone.0350110)
Supplement: Attachment — Submitted filename: Response to reviewers.docx [file pone.0350110.s004.docx]

Dear Dr. Pinzon,

We thank you for your effort to assess our manuscript. The feedback on the manuscript was helpful and we feel that this contributed to improve the quality of our paper.

In response to the comments, we have revised the manuscript accordingly, with all changes highlighted using track changes. We have also provided a detailed, point-by-point reply addressing each concern.

Key revisions include a clearer presentation of the study’s aim and methods to enhance replicability. As requested, we have restructured the discussion to first elaborate on the main findings. Additionally, we have expanded the limitations section.

We have also made the necessary adjustments to meet the journal's requirements, including updating our funding statement: ‘In 2017, DRAIM was developed as a pilot project with funding from the "Stichting Kwaliteitsgelden Medisch Specialisten" (SKMS). However, funding from SKMS ceased at the end of 2022. Since 2023, DRAIM has been supported by Zorgverzekeraars Nederland (ZN). No additional external funding was received for this study.’

We believe that the revised manuscript is now suitable for publication in *PLOS ONE*.

On behalf of all authors,

M.G.A.M. van der Velde & E.M. Mols

**Point to point response**

***Reviewer#1***

Thank you authors for the submission. 
This study is very interesting. 
There is novelty in this study.   
I give some comments and suggestions.

*We thank you for your effort to assess our manuscript. The feedback on the manuscript was helpful and we feel that this helped us to improve the quality of our paper*

Comment 1.1: The method should be clear to replicate the findings.

*Reply 1.1: Thank you for your comment. We agree and have revised the manuscript to improve clarity and ensure replicability of the findings. The most significant change is that we reorganized the text to follow a more standard structure: Introduction, Methods, Results, Discussion, and Conclusion. In particular, we have rewritten the Methods section for better clarity, added the statistical methods, and ensured that all methodological content is now included in this section, rather than in the previous "Findings to Date" section. These revisions should facilitate replication of the findings. Additionally, we have rewritten the Results section to focus solely on the results, moving any introductory content to the Introduction section.*

*See page 4, lines 76-81 for the adjustment of the introduction: ‘Additionally, in contemporary emergency care, an increasing proportion of the population consists of undifferentiated patients, characterized by older age, comorbidity, polypharmacy and non-specific complaints. These patients often visit the ED due to exacerbations of chronic conditions and are typically treated by an internist. In 2018, they represented 20% of all patients in Dutch EDs; a percentage that is expected to rise considering the ageing population and increasing prevalence of chronic diseases (6).*

*See page 5, lines 99-117, for adjustments to the method section: ‘The Máxima Medical Centre was the first participating hospital to have fully automated its data-extraction and submission. Therefore, we used the data of this hospital as a showcase with a retrospective single-center cohort study design. Máxima MC – Veldhoven is a teaching hospital in the Netherlands. The ED of the participating hospital contains 15 treatment bays, including one trauma-room and one shock-room. An Acute Medical Unit is present.* *Eligible patients for inclusion in the quality registry were all patients who presented or were admitted to the specialty of internal medicine from the ED, including its subspecialties such as infectious diseases, vascular medicine, geriatric care, endocrinology, nephrology, acute medicine, hematology, oncology, immunology and allergology. Patients under the age of 18 years were excluded.*

*For this study, all consecutive patients aged ≥65 years who presented to the ED with internal medicine-related complaints were included. Patients were stratified into three age categories: 65–74 years, 75–84 years, and ≥85 years. To distinguish between patients with specific versus nonspecific presentations, we categorized patients with the primary presenting complaint general malaise as non-specific and all other primary presenting complaints as specific. . While general malaise is not strictly synonymous with the definition of nonspecific complaints found in the literature, we assumed that a considerable proportion of these patients would fit the NSC criteria defined by Nemec et al, due to the absence of a more specific presenting complaint (1).’*

*See page 8-9, lines 151-168: Statistical method. Baseline characteristics of the participants were analyzed using descriptive statistics. Continuous data were presented as mean and standard deviation (SD) or median and interquartile range (IQR), and nominal data were presented as total amount and percentage. To compare the baseline characteristics between groups, p-values were calculated by using Pearson’s chi-square or Fisher’s exact test for categorical variables, and the independent-samples t-test or Mann-Whitney U test for continuous variables. A significance level of 5% was used for all statistical tests. Statistical analyses were performed using the software Statistical Package for the Social Sciences (SPSS), version 27 (IBM Corp, New York, USA). Patient and public involvement. A representative from the Dutch Patient Federation is involved in the development of DRAIM to ensure patient representation. Ethics. The collection of the data as described above has been approved by the Medical Ethical Committee of the Máxima MC. A waiver for informed consent was obtained because of the observational nature of the project.’*

Comment 1.2: The registry should include exact diagnosis (based on ICD), co-morbidity, treatment, and outcome.  
*Reply 1.2: Thank you for your feedback. As stated in the article, our data is sourced from the Dutch Registry for Acute and Internal Medicine (DRAIM). The methods section provides a comprehensive list of the recorded variables (Table 1). Diagnoses are based on ICD-10 classification, which we have now clarified in Table 1, including reference* (2).*Additionally, comorbidities are collected and provided as an age-adjusted Charlson Comorbidity Index. Furthermore, outcomes are included in the registry, i.e. mortality, hospital admission, ED-revisits, hospital readmission and ICU admission. Currently, the DRAIM registry does not capture information on treatments received in the emergency department (ED). Given the complexity of acute conditions and the wide range of possible treatments, it is not feasible to collect and analyze this data comprehensively at this stage. However, as DRAIM continues to develop, we will assess whether treatment details and adherence to guidelines can be incorporated into the registry. We have clarified this in the limitations section—please refer to pages 17 lines 317-321 for the updated text: ‘Furthermore, the registry does not capture information on specific treatments received in the ED. Recording all treatments in a standard quality registry is not feasible due to the vast number and complexity of treatment options, as well as the lack of standardized documentation in EHRs. However, as DRAIM continues to develop, we will assess whether treatment details and adherence to guidelines can be incorporated into the registry’.*

 Comment 1.3: Is it too broad to include all diagnosis in internal medicine? The board criteria will not focus in the specific treatment and outcome.

*Reply 1.3: Thank you for your comment. As mentioned in our response above, the DRAIM registry does includes all ICD-10 diagnoses within internal medicine, but it does not yet capture treatment details for these conditions. Despite that we did not report ICD-10 diagnosis, the quality registry does allow for analysis of diagnoses using either ICD-10 codes or presenting complaints.*

Comment 1.4: Please begin the discussion with the main findings of your study.

*Reply 1.4: We agree with your comment and have revised the discussion to begin with the main findings of our study.* See pages *14-15, lines 248-253: This DRAIM-cohort study shows that approximately half of the patients presenting for internal medicine in the ED were aged ≥65 years, and one-third presented with general malaise. Patients with general malaise were more often older, female, had more comorbidities, and were triaged into lower urgency categories. Additionally, they experienced more adverse outcomes, including longer LOS-ED, higher hospital admission rates, but lower ICU admission rates.’*

Comment 1.5: Please elaborate more the limitation of the study.

*Reply 1.5: Thank you for your comment. We have expanded the limitations section to provide a more detailed discussion, as mentioned in our previous responses. Specifically, we have clarified the constraints of the DRAIM registry, including the lack of treatment data and the broad scope of internal medicine diagnoses. Additionally, we have outlined future plans for more focused analyses of specific diseases and their treatments. Please see the revised limitations section on pages 17, lines 308-323: ‘Limitations of our study include the following: firstly, a minority of variables in this cohort were incomplete. However, we try to minimize incomplete variables, by providing the local data collection team with feedback reports on missing data. Secondly, data extraction relies on the data registry in the electronic health records (EHR), where diagnoses are primarily based on financial records. This approach can lead to inaccuracies, as seen in cases where patients presenting with acute exacerbations of chronic conditions may be classified under their underlying chronic illness rather than the acute episode itself. Similarly, in the center used for this cohort, patients referred to the ED by their primary medical specialist were registered as self-referral, making it difficult to distinguish them from true self-referred patients. Additionally, while the registry uses ICD-10 coding, we have further grouped diagnoses into broader categories to facilitate meaningful data analysis. This aids the identification of trends, but may also contribute to a loss of diagnostic granularity. Furthermore, the registry does not capture information on specific treatments received in the ED. Recording all treatments in a standard quality registry is not feasible due to the vast number and complexity of treatment options, as well as the lack of standardized documentation in EHRs. However, as DRAIM continues to develop, we will assess whether treatment details and adherence to guidelines can be incorporated into the registry. Lastly, data is gathered and analyzed with a delay of at least one month. Providing real-time insights would enhance the value of the reported information, allowing for immediate adjustments in the organization of care.*

Comment 1.6: The conclusion should be concise and clear, and only answer the research question.

*Reply 1.6: We agree with your comment and have revised the conclusion, adding a subheading to enhance clarity and ensure it directly answers the research question. See pages 17-18, lines 326-329:* *DRAIM provides valuable insights and identifying bottlenecks in the patient journey throughout the acute care pathway, facilitating the evaluation and improvement of acute care quality. We have demonstrated that older patients presenting to the ED with poorly defined symptoms experience more adverse outcomes, particularly longer LOS-ED and higher hospital admission rates.*

1.	Nemec M, Koller MT, Nickel CH, Maile S, Winterhalder C, Karrer C, et al. Patients presenting to the emergency department with non-specific complaints: the Basel Non-specific Complaints (BANC) study. Acad Emerg Med. 2010;17(3):284-92.

2.	World Health O, Pan American Health O. The ICD-10 classification of mental and behavioural disorders: Diagnostic criteria for research. 1993.
