# Supplementary material for: Introducing the Dutch Quality Registry for Acute Internal Medicine (DRAIM): Method of development and opportunities of use from a single-centre pilot study
Source: PLoS One. 2026 Jun 1;21(6):e0350110. doi: 10.1371/journal.pone.0350110 (PMC13225412; doi:10.1371/journal.pone.0350110)
Supplement: Attachment — Submitted filename: Response to reviewers 2.docx [file pone.0350110.s005.docx]

Dear Editor,

We apologize for the numbering error in our previous submission. Table 3 was inadvertently omitted due to a clerical oversight in the sequence; however, please note that no new data has been added. We have simply corrected the numbering throughout the manuscript to ensure consistency.

Kind regards,

Marleen van der Velde
